# Real-world validation of ECOG performance status and neutrophil-to-lymphocyte ratio in second-line paclitaxel plus ramucirumab for advanced gastric cancer

**DOI:** 10.1515/biol-2025-1191

**Published:** 2026-02-24

**Authors:** Hong Cheng, Lulu Sun, Xinyue Wang, Yixia Wang, Yu Chen, Kejin Cai, Xiaoli Hou

**Affiliations:** Institute of Translational Medicine, School of Medicine, Yangzhou University, Yangzhou, Jiangsu 225001, China; The Key Laboratory of the Jiangsu Higher Education Institutions for Integrated Traditional Chinese and Western Medicine in Senile Diseases Control, Yangzhou University, Yangzhou, Jiangsu 225001, China; The Key Laboratory of the Jiangsu Higher Education Institutions for Nucleic Acid & Cell Fate Regulation, Yangzhou University, Yangzhou, Jiangsu 225001, China; Department of Medical Science, Yangzhou Polytechnic University, Yangzhou, Jiangsu 225000, China

**Keywords:** advanced gastric cancer, paclitaxel, ramucirumab, overall survival, progression-free survival, prognostic factors

## Abstract

Advanced gastric cancer (AGC) has a poor prognosis; better second-line options are needed. We retrospectively reviewed 130 AGC patients treated at one center with paclitaxel 80 mg/m^2^ (days 1, 8, 15) plus ramucirumab 8 mg/kg (days 1, 15) every 28 days after failure of platinum/fluoropyrimidine therapy. Kaplan–Meier curves estimated overall (OS) and progression-free survival (PFS); Cox models identified prognostic factors. Median OS was 8.3 months (95 % CI 6.9–9.7) and median PFS 4.2 months (95 % CI 3.3–5.1); 12-month OS was 31.8 %. Objective response and disease-control rates were 19.2 % and 50.0 %, respectively. Grade ≥ 3 toxicity occurred in 33 % of patients, mainly neutropenia (19 %) and neuropathy (14 %). Multivariable analysis linked longer OS to ECOG 0–1 (HR 0.54, *p* = 0.011) and a low neutrophil-to-lymphocyte ratio (HR 0.59, *p* = 0.017). In this real-world single-center cohort, paclitaxel plus ramucirumab provided clinically meaningful benefit with manageable toxicity. ECOG performance status and NLR confirmed their prognostic value in this real-world cohort. Further multicenter studies may refine patient selection and optimize outcomes. Clinically, ECOG and NLR can be used to communicate prognosis, tailor follow-up/supportive care, and stratify patients in routine practice receiving paclitaxel–ramucirumab.

## 1 Introduction

Advanced gastric cancer (AGC) remains a major global health burden: it is the fifth most commonly diagnosed malignancy and the fourth leading cause of cancer-related death worldwide, with mortality largely driven by late-stage presentation and suboptimal therapeutic efficacy [1], 2]. First-line systemic therapy typically consists of a platinum–fluoropyrimidine doublet; however, despite widespread use, these regimens yield only modest response rates and limited improvements in overall survival [2], 3]. Second-line therapy, such as paclitaxel combined with ramucirumab and irinotecan-based regimens have demonstrated improved outcomes with the paclitaxel-ramucirumab combination [1], 4]. However, challenges remain in predicting patient responses and optimizing treatment strategies [4], 5].

The predictive value of inflammatory and tumor biomarkers for treatment efficacy in AGC remains uncertain, highlighting the need for robust, externally validated predictors to enable individualized therapy [1]. Integrating clinical variables with biomarker-based prognostic indices may improve risk stratification, identify patients most likely to benefit from targeted approaches, and facilitate more efficient allocation of healthcare resources [6], 7]. Addressing these evidence gaps is essential to advance therapeutic decision-making and outcomes in advanced gastric cancer.

While paclitaxel plus ramucirumab has shown promise in second-line settings, robust practice-based data on prognostic factors remain limited. We therefore aimed to (i) describe real-world effectiveness and safety of second-line paclitaxel plus ramucirumab and (ii) validate the prognostic relevance of ECOG performance status and NLR within this treatment context, thereby complementing existing trial and registry evidence.

## 2 Methods and materials

### 2.1 Study Design and Setting

This was a real-world, retrospective study conducted at the School of Medicine, Yangzhou University from January 2020 to December 2022.

**Informed consent:** Informed consent has been obtained from all individuals included in this study.

**Ethical approval:** The research related to human use has been complied with all the relevant national regulations, institutional policies and in accordance with the tenets of the Helsinki Declaration, and has been approved by the Ethics Committee of the School of Medicine, Yangzhou University (YXYLL-2023088).

### 2.2 Patient Eligibility

*Inclusion Criteria*: 1), Histologically confirmed advanced gastric or gastroesophageal junction adenocarcinoma; 2), Progression on or intolerance to first-line chemotherapy (typically a platinum/fluoropyrimidine regimen); 3), Received ≥1 cycle of second-line therapy with paclitaxel plus ramucirumab at our center; 4), Age ≥18 years; 5), Adequate organ function at the start of second-line therapy (e.g., absolute neutrophil count ≥1.5 × 10^9^/L, platelets ≥100 × 10^9^/L, serum creatinine ≤1.5 × upper limit of normal).

*Exclusion Criteria*: 1), a concurrent malignancy requiring active treatment, except non-melanoma skin cancer; 2), concurrent enrollment in another interventional trial of an investigational agent for second-line therapy; 3), severe or uncontrolled comorbidities for which paclitaxel or ramucirumab was contraindicated (e.g., decompensated heart failure, refractory hypertension); 4), in the retrospective cohort, incomplete medical records that precluded extraction of prespecified variables.

## 3 Treatment protocol

*Drug Administration*: Paclitaxel was administered at 80 mg/mˆ2 on Days 1, 8, and 15 of each 28-day cycle. Ramucirumab was administered at 8 mg/kg on Days 1 and 15 of each 28-day cycle. Dose modifications – including reductions or delays – were implemented per institutional guidelines on the basis of hematologic and non-hematologic toxicities graded according to the Common Terminology Criteria for Adverse Events (CTCAE), version 5.0.

*Supportive Care*: Antiemetics, granulocyte colony-stimulating factor (G-CSF), and other supportive measures were provided at the treating physician’s discretion in accordance with institutional practice. Hypertension was managed by optimizing baseline blood pressure and initiating or adjusting antihypertensive therapy as clinically indicated.

### 3.1 Sample size calculation

An *a priori* target enrollment of 100–130 patients was selected based on feasibility and anticipated event rates. Under a proportional hazards framework (Schoenfeld approximation), this sample was projected to provide ≥80 % power at a two-sided *α* = 0.05 to detect a hazard ratio of approximately 0.60 for prespecified prognostic covariates in the Cox model, accounting for anticipated censoring.

### 3.2 Statistical analysis

Continuous variables were summarized as mean ± SD or median (range), as appropriate, and categorical variables as counts (percentages). Overall survival (OS) and progression-free survival (PFS) were estimated using the Kaplan–Meier method, with between-group comparisons by the log-rank test. Univariable Cox proportional hazards models were fitted for each candidate predictor; variables with p < 0.10 in univariable analyses, together with clinically relevant covariates, were entered into multivariable Cox models. Effect estimates are reported as hazard ratios (HRs) with 95 % confidence intervals (CIs), and two-sided p < 0.05 was considered statistically significant. Assumptions of proportional hazards were. Candidate baseline covariates were specified *a priori* from clinical plausibility and availability at the start of second-line therapy: age, sex, ECOG performance status (0–1 vs ≥ 2), comorbidity burden (CCI ≥2), tumor location (GEJ vs gastric), histology (diffuse vs others), peritoneal metastasis, response to first-line therapy (CR/PR vs SD/PD), PD-L1 expression, laboratory/biomarker indices (albumin, CRP, CEA, CA19-9, NLR, PLR). We did not treat post-baseline treatment-delivery variables (e.g., dose intensity, number of cycles) as baseline predictors in the primary multivariable model to avoid immortal-time bias, although they are described in univariable summaries. Variable entry followed a pre-specified scheme: we forced in ECOG PS and NLR (clinical *a priori* interest), then included any additional covariates with univariable p < 0.10. To prevent over-fitting, we capped model size to maintain ≥10 events per parameter. Proportional-hazards assumptions were evaluated using Schoenfeld residuals and log(–log) plots; multicollinearity was screened with variance inflation factors. Analyses used complete-case data with two-sided p < 0.05 considered significant.

Survival was summarized with Kaplan–Meier methods; medians and time-point estimates include 95 % confidence intervals (CIs) using Greenwood’s formula. Group comparisons used the log-rank test. We displayed number-at-risk tables beneath KM curves. Multivariable associations with OS were estimated with Cox proportional hazards models; proportional-hazards assumptions were evaluated using Schoenfeld residuals and log(–log) plots. Forest plots depict hazard ratios (HRs) with 95 % CIs from the final multivariable model.

We performed exploratory subgroup analyses by prior checkpoint inhibitor exposure (any prior PD-1/PD-L1/CTLA-4 vs none) and prior trastuzumab (any prior exposure vs none). We compared KM medians (OS/PFS) and time-point survival (6/12 months) and estimated adjusted hazard ratios using Cox models controlling for age, sex, ECOG (0–1 vs ≥ 2), and NLR (<3 vs ≥ 3). For trastuzumab, we conducted a HER2-positive–only sensitivity analysis. ORR differences were evaluated with logistic regression using the same covariates.

All analyses were conducted using R (version 4.4), with a p-value <0.05 considered statistically significant.

## 4 Results

### 4.1 Baseline patient characteristics

The cohort comprised 130 patients (Table 1). The median age was 64 years (range, 36–82), and 60 % were male. Most patients had an Eastern Cooperative Oncology Group (ECOG) performance status (PS) of 0–1 (70 %), whereas 30 % had PS ≥ 2 (Table 1). Primary tumors were predominantly located in the gastric body or antrum (77 %), and, by Lauren classification, 54 % were of the intestinal subtype (Table 1). Platinum/fluoropyrimidine regimens were the predominant first-line therapy (88 %), and 38 % of patients had achieved a partial or complete response prior to second-line therapy (Table 1).

**Table 1:** Baseline patient characteristics

Variable	Value
**Age, years**	Median 64 (range 36–82)
**Sex**	
Male	78 (60.0 %)
Female	52 (40.0 %)
**ECOG performance status**	
0	25 (19.2 %)
1	66 (50.8 %)
2	31 (23.8 %)
≥3	8 (6.2 %)
**Tumor location**	
Gastric (body/antrum/fundus)	100 (76.9 %)
Gastroesophageal junction	30 (23.1 %)
**Histological subtype**	
Intestinal	70 (53.8 %)
Diffuse	48 (36.9 %)
Mixed/Other	12 (9.2 %)
**Prior first-line therapy**	
Platinum/fluoropyrimidine	115 (88.5 %)
Other regimens	15 (11.5 %)
**Response to First-line Therapy**	
CR or PR	50 (38.5 %)
SD	35 (26.9 %)
PD	45 (34.6 %)
**Comorbidity burden** (CCI ≥2)	*n* = 45 (34.6 %)
**HER2 (IHC 3+ or ISH-amplified)**	34 (26.2 %)
**PD-L1 (CPS ≥1)**	57 (43.8 %)
**Mismatch-repair deficiency/MSI-high**	11 (8.5 %)

ECOG, eastern cooperative oncology group; CCI, charlson comorbidity index; CR, complete response; PR, partial response; SD, stable disease; PD, progressive disease.

## 5 Treatment delivery and efficacy outcomes

**Figure 1:**
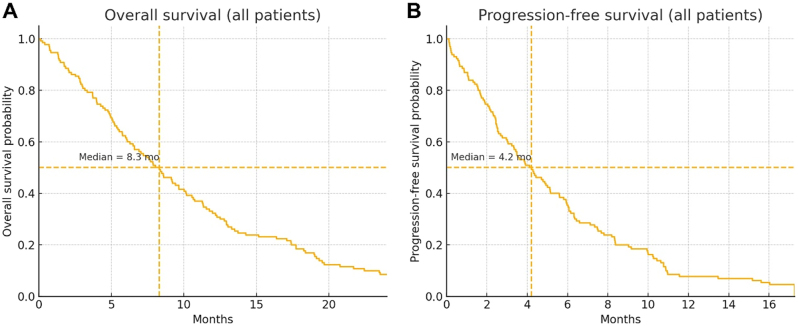
Kaplan–Meier survival curves. (A) Overall survival for the entire cohort (*n* = 130). The dashed lines indicate the median OS 8.3 months; the 6-month and 12-month OS rates were 54.6 % and 31.8 %, respectively. (B) Progression-free survival for the entire cohort with median PFS 4.2 months..

The median number of cycles received was five (range, 1–15), and 21.5 % of patients required at least one dose reduction (Table 2). The median overall survival (OS) for the entire cohort was 8.3 months (95 % CI, 6.9–9.7), and the median progression-free survival (PFS) was 4.2 months (95 % CI, 3.3–5.1) (Table 2 and Figure 1A and B). The 12-month OS rate was approximately 30 % (Table 2). An objective response rate of 19.2 % was observed among those evaluated radiologically, and the disease control rate was 50.0 % (Table 2).

**Table 2:** Treatment delivery and efficacy outcomes.

Parameter	Value
Median no. of treatment cycles (range)	5 (1–15)
Dose reductions required	28 (21.5 %)
Dose delays >1 week	18 (13.8 %)
Median overall survival, months	8.3 (95 % CI: 6.9–9.7)
Median progression-free survival, months	4.2 (95 % CI: 3.3–5.1)
6-Month OS rate	71 (54.6 %)
12-Month OS rate	40 (31.8 %)
Objective response rate	25 (19.2 %)
Disease control rate	65 (50.0 %)

## 6 Adverse events

Grade ≥3 adverse events occurred in 33 % of patients. The most frequent high-grade events were neutropenia (19 %) and peripheral neuropathy (14 %) (Table 3). Any-grade hypertension and fatigue were observed in 17 % and 23 % of patients, respectively, and were predominantly grade 1–2. Proteinuria was documented in 5.4 % of patients overall, including 2.3 % with grade ≥3 events (Table 3).

**Table 3:** Adverse events.

Adverse event	All grades, *n* (%)	Grade ≥3, *n* (%)
Neutropenia	32 (24.6 %)	25 (19.2 %)
Neuropathy (sensory)	26 (20.0 %)	18 (13.8 %)
Hypertension	22 (16.9 %)	8 (6.2 %)
Fatigue	30 (23.1 %)	10 (7.7 %)
Gastrointestinal toxicity (e.g., diarrhea)	18 (13.8 %)	5 (3.8 %)
Proteinuria	7 (5.4 %)	3 (2.3 %)

Overall incidence of grade ≥3 AEs: 43 (33 %).

### 6.1 Prognostic factor analysis for overall surviva

**Figure 2:**
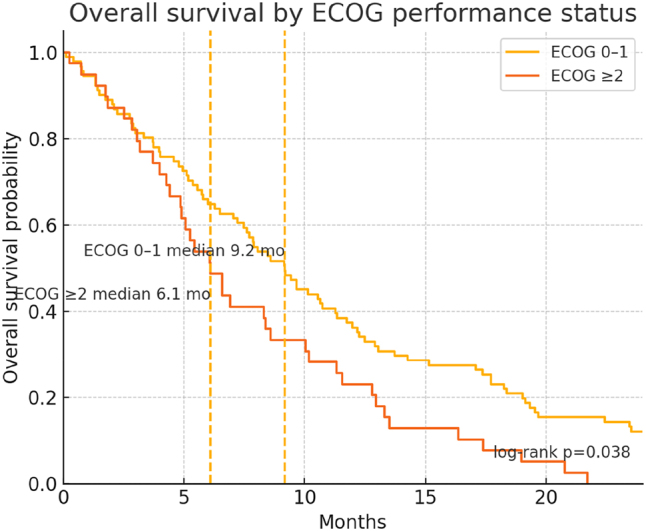
Overall survival by ECOG performance status. KM curves for ECOG 0–1 (*n* = 91) versus ECOG ≥2 (*n* = 39). Median OS was 9.2 months for ECOG 0–1 and 6.1 months for ECOG ≥2; groups differed significantly by log-rank test (*p* = 0.038). Dashed verticals mark group medians.

**Figure 3:**
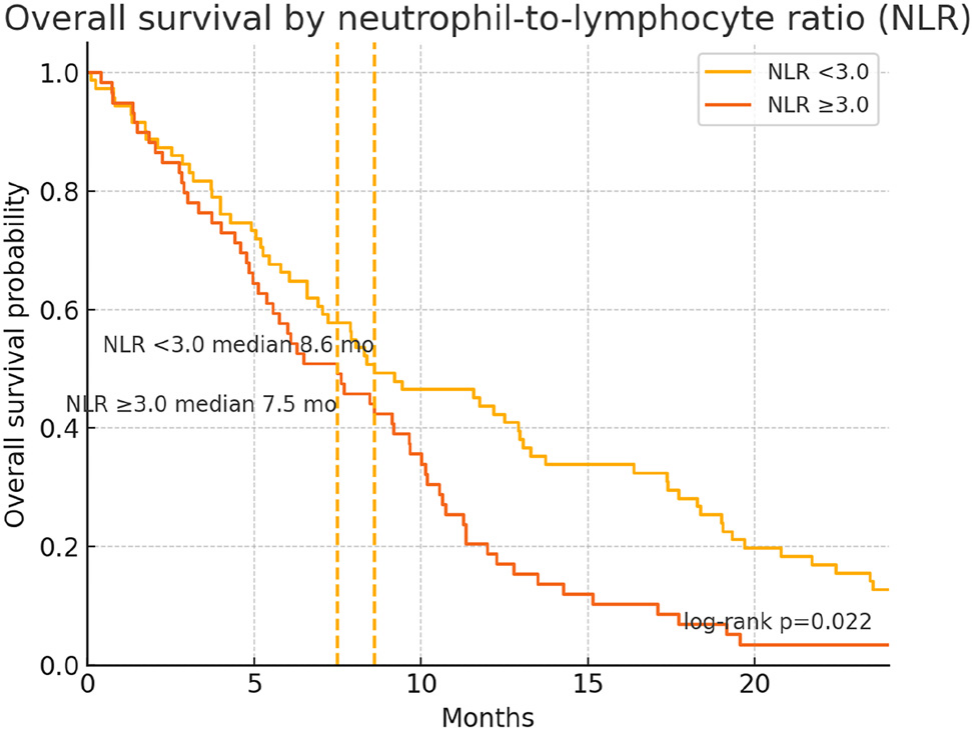
Overall survival by neutrophil-to-lymphocyte ratio (NLR). KM curves for NLR <3.0 (*n* = 71) versus NLR ≥3.0 (*n* = 59). Median OS was 8.6 months and 7.5 months, respectively; log-rank *p* = 0.022. Dashed verticals mark group medians. These curves complement the independent association of lower NLR with longer OS in the multivariable model.

Table 4 presents the univariable and multivariable Cox regression models identifying factors associated with OS. On univariable analysis, ECOG PS 0–1 and a low neutrophil-to-lymphocyte ratio (NLR) correlated significantly with prolonged survival (*p* = 0.004 and *p* = 0.030, respectively) (Table 4, Figures 2 and 3). In the multivariable model, both ECOG PS 0–1 (HR 0.54, *p* = 0.011) and low NLR (HR 0.59, *p* = 0.017) remained independently predictive of better OS (Table 4, Figures 2 and 3). Other variables, including age, sex, tumor location, and CA19-9 levels, did not achieve statistical significance in the final adjusted model (Table 4).

**Table 4:** Prognostic factor analysis for overall survival.

Factor	Univariable HR (95 % CI)	p-Value	Multivariable HR (95 % CI)	p-Value
**Demographic factors**				
Age (≥65 vs. <65 years)	1.23 (0.82–1.84)	0.30	–	–
Sex (female vs. male)	0.95 (0.60–1.51)	0.82	–	–
Body mass index (BMI≥25 vs. <25 kg/m^2^)	0.88 (0.54–1.42)	0.61	–	–
**Clinical & disease-related factors**				
ECOG PS (0–1 vs. ≥2)	0.50 (0.31–0.80)	0.004	0.54 (0.35–0.83)	0.011
Comorbidity burden (CCI ≥2 vs. <2)	1.28 (0.85–1.93)	0.24	–	–
Tumor location (GE junction vs. gastric)	1.10 (0.67–1.79)	0.71	–	–
Histological subtype (diffuse vs. others)	1.20 (0.79–1.83)	0.39	–	–
Peritoneal metastasis (yes vs. no)	1.25 (0.82–1.91)	0.29	–	–
Prior response to 1 L Tx (CR/PR vs. SD/PD)	0.89 (0.56–1.40)	0.61	–	–
**Treatment-related factors**				
Dose intensity (≥80 % vs. <80 % of planned)	0.76 (0.49–1.17)	0.21	–	–
Number of cycles (≥6 vs. <6)	0.70 (0.43–1.12)	0.14	–	–
**Laboratory & biomarker factors**				
NLR (<3.0 vs. ≥3.0)	0.62 (0.40–0.96)	0.030	0.59 (0.38–0.92)	0.017
PLR (<150 vs. ≥150)	0.82 (0.53–1.27)	0.39	–	–
Albumin (>3.5 vs. ≤3.5 g/dL)	0.70 (0.44–1.11)	0.13	–	–
CRP (<5 mg/L vs. ≥5 mg/L)	1.20 (0.76–1.89)	0.45	–	–
CEA (≤5 vs. >5 ng/mL)	1.15 (0.74–1.79)	0.54	–	–
CA19-9 (≤37 vs. >37 U/mL)	1.21 (0.77–1.90)	0.40	–	–
PD-L1 expression (positive vs. negative)	0.85 (0.51–1.40)	0.51	–	–

Multivariable model specification: ECOG PS and NLR forced in (pre-specified), plus any baseline covariates with univariable p < 0.10; post-baseline treatment-delivery variables were excluded from multivariable modeling to avoid immortal-time bias. Proportional-hazards assumptions evaluated with Schoenfeld residuals; VIFs screened for multicollinearity. ^1^Pre-specified a binary threshold of NLR <3.0 versus ≥ 3.0 was based on prior literature showing common use of 3.0 (median cut-off close to three across gastrointestinal-cancer meta-analyses) and robust prognostic associations across a range of cut-offs in gastric cancer.

Outcomes did not differ significantly by HER2, PD-L1 (CPS ≥1), or MSI-H status after adjustment for baseline covariates and relevant prior therapies (all p > 0.05). Point estimates were near unity, and CIs were wide for MSI-H reflecting small numbers (*n* = 11) (Supplementary Table S1).

### 6.2 Subgroup analysis by ECOG performance status

Exploratory analyses showed no statistically significant differences in OS or PFS by prior ICI exposure (11 % of cohort) or prior trastuzumab (14 %). Effect estimates were near unity after adjustment for age, sex, ECOG, and NLR (Supplementary Table S2). Findings were similar when restricting the trastuzumab analysis to HER2-positive patients (Supplementary Table S3).

Patients with PS 0 had the longest median OS of 11.2 months, compared to 8.5, 5.8, and 3.2 months for those with PS 1, 2, and ≥3, respectively (Table 5). This gradient persisted in PFS analyses, highlighting the critical prognostic impact of functional status in advanced gastric cancer treated with paclitaxel plus ramucirumab.

**Table 5:** Subgroup analysis by ECOG performance status.

ECOG PS	*n* (%)	Median OS, months (95 % CI)	Median PFS, months (95 % CI)	HR for OS (reference = ECOG 0) [95 % CI]	p-Value*
0	25 (19.2 %)	11.2 (8.8–13.6)	5.8 (4.2–7.4)	1.00 (reference)	–
1	66 (50.8 %)	8.5 (6.9–10.1)	4.3 (3.2–5.2)	1.35 (0.85–2.15)	0.19
2	31 (23.8 %)	5.8 (4.1–7.5)	3.2 (2.3–4.1)	2.01 (1.25–3.25)	0.002
≥3	8 (6.2 %)	3.2 (2.1–4.3)	2.1 (1.3–3.0)	3.11 (1.30–4.65)	0.001

*p-Value obtained from a Cox proportional hazards model comparing each ECOG subgroup to the ECOG 0 reference group.

## 7 Discussion

In this real-world cohort, second-line paclitaxel plus ramucirumab achieved outcomes consistent with expectations, and ECOG performance status together with NLR confirmed their prognostic value. These findings complement prior evidence by demonstrating applicability in routine practice, including patients with poorer performance status and greater comorbidity burden. Toxicities were generally manageable and consisted mainly of neutropenia and mild-to-moderate neuropathy. By analyzing a consecutive, unselected cohort treated outside of clinical trials, we provide complementary evidence that ECOG PS and NLR retain prognostic discrimination in everyday care, thereby supporting their routine use for risk communication and follow-up planning.

In AGC, performance status, systemic inflammatory markers, and tumor-derived biomarkers are increasingly used to guide risk stratification and individualized therapy. Composite indices – such as an inflammatory burden index combining C-reactive protein (CRP) and the neutrophil-to-lymphocyte ratio (NLR) – have been linked to postoperative complications and mortality in patients receiving neoadjuvant therapy [8], 9]. Simpler ratios, including NLR and the platelet-to-lymphocyte ratio (PLR), correlate with tumor stage and chemotherapy responsiveness [10], 11]. More comprehensive scores (e.g., the prognostic immune-inflammatory index) predict both progression-free and overall survival [12]. In parallel, molecular profiling – encompassing CDH1 alterations, circulating interleukin-6 (IL-6), and tumor-microenvironment signatures – can further refine risk assessment and inform the selection of anti-angiogenic and immunotherapeutic strategies [13]. Integrating these laboratory markers with clinical factors and actionable molecular targets (HER2, PD-L1, mismatch-repair status) may improve treatment selection and outcomes [14], 15]. Nonetheless, rigorous external validation in ethnically and geographically diverse populations remains essential, as genetic background and treatment context may modulate biomarker performance [9].

In our cohort, 11 % of patients had previously received an immune checkpoint inhibitor and 14 % had received trastuzumab. In exploratory analyses, prior exposure to either agent was not associated with differential efficacy of subsequent paclitaxel–ramucirumab therapy (data not shown), consistent with prior real-world reports [16], 17]. Although trastuzumab plus paclitaxel is active in HER2-positive disease, prior biologic therapy has not consistently improved outcomes with later VEGFR-2–targeted treatment [18], 19]. Larger, adequately powered studies are needed to define optimal sequencing strategies [16].

The second-line landscape for AGC has evolved rapidly. The RAINBOW trial established ramucirumab plus paclitaxel as superior to paclitaxel alone [20]. Subsequent phase II data for ramucirumab plus docetaxel showed an objective response rate of 25.7 % and a disease control rate of 74.3 % [21]. Prior exposure to nivolumab has been associated with improved progression-free survival on subsequent taxane-based therapy [22]. Emerging combinations-such as fruquintinib plus sintilimab and sintilimab plus nab-paclitaxel-also demonstrate encouraging activity, particularly in immunotherapy-naïve populations [23], 24]. Moreover, the addition of checkpoint blockade to ramucirumab can outperform chemotherapy alone, and real-world evidence supports the tolerability and effectiveness of paclitaxel-ramucirumab [20], 25]. Collectively, these findings support a biomarker-driven, personalized approach that integrates inflammatory and immune markers with clinicopathologic features.

This study has several limitations. It was conducted at a single center with a partly retrospective design, which may limit external validity and introduce selection and information biases. The modest sample size and variable follow-up reduce statistical power and the precision of effect estimates, particularly for subgroup analyses. Residual and unmeasured confounding cannot be excluded. Prospective, multicenter studies with harmonized endpoints and pre-specified, standardized biomarker collection are needed to validate these observations and to refine integrated risk models that combine clinical, inflammatory, and molecular predictors.

Systemic inflammation can promote tumor growth and immune evasion via cytokine (e.g., IL-6/STAT3) and myeloid-cell programs, while lymphocyte depletion reflects impaired adaptive immunity [26]. As a readily obtainable composite of neutrophilia and lymphopenia, NLR acts as a practical surrogate for this tumor–host balance and has shown consistent prognostic associations across solid tumors and in gastric cancer cohorts [27], 28]. In our practice-based series – including patients under-represented in trials – NLR’s signal persisted, suggesting that inflammatory tone captured at baseline retains clinical meaning when decisions are made in routine care.

VEGF/VEGFR2 signaling not only drives aberrant vasculature but also shapes an immunosuppressive microenvironment by limiting T-cell trafficking and directly modulating effector and regulatory T cells [29]. Anti-VEGF/VEGFR2 therapy can transiently normalize tumor vessels, improving perfusion, drug delivery, and immune cell infiltration [30], 31]. Ramucirumab, a high-affinity monoclonal antibody to VEGFR2, blocks ligand–receptor binding and attenuates angiogenic signaling [32]. Within this framework, a lower inflammatory burden (lower NLR) may coincide with a microenvironment more amenable to vascular normalization and chemotherapy delivery, offering a plausible biological lens for the observed survival gradients.

Clinically, ECOG PS and NLR can support risk communication, triage for closer follow-up and supportive care, and stratification/enrichment in future trials of anti-angiogenic regimens and combinations. Translationally, prospective work should pair baseline NLR with soluble inflammatory markers (e.g., CRP, IL-6), myeloid-derived suppressor cell phenotyping, and tissue immune profiling, to test whether inflammatory tone modifies the depth or duration of benefit from VEGFR2 blockade and taxanes.

This analysis was conducted at a single center in China with a partly retrospective design and a practice-based case-mix, which may limit generalizability to other geographies and health-system settings. Differences in patient selection, distribution of biomarkers, supportive-care norms, and availability of subsequent lines of therapy could influence outcomes and attenuate transportability to centers with different resources or trial-like populations. Although dosing, schedules, and toxicity management followed widely used standards, and our observed OS and PFS align with expectations for this regimen, these results should be interpreted as real-world validation that complements rather than replaces multi-center evidence. Future prospective, multi-center studies – ideally with predefined biomarker collection and harmonized post-protocol therapy capture – are needed to confirm external validity.

Second-line paclitaxel plus ramucirumab provides clinically meaningful benefit in AGC with an acceptable safety profile. Performance status and readily obtainable inflammatory indices – particularly the neutrophil-to-lymphocyte ratio – offer incremental prognostic value and can help clinicians anticipate disease course and individualize supportive care. External validation in larger, prospectively characterized cohorts is warranted to define their role in routine decision-making.

## Supplementary Material

Supplementary Material
