# Supplementary material for: Real-world validation of ECOG performance status and neutrophil-to-lymphocyte ratio in second-line paclitaxel plus ramucirumab for advanced gastric cancer
Source: Open Life Sci. 2026 Feb 24;21(1):20251191. doi: 10.1515/biol-2025-1191 (PMC12927453; doi:10.1515/biol-2025-1191)
Supplement: Supplementary file 1 — Supplementary Material [file j_biol-2025-1191_suppl_001.docx]

**Supplementary Table S1. Outcomes by baseline biomarker status**

| **Group** | **n** | **Median OS, mo (95% CI)** | **Median PFS, mo (95% CI)** | **12‑mo OS (%)** | **ORR n/N (%)** | **Adjusted HR for OS† (95% CI); p** | **Adjusted HR for PFS† (95% CI); p** | **Adjusted OR for ORR‡ (95% CI); p** |
| --- | --- | --- | --- | --- | --- | --- | --- | --- |
| **HER2 status** |  |  |  |  |  |  |  |  |
| **HER2‑negative** | 96 | 8.3 (6.9–9.8) | 4.2 (3.3–5.1) | 31.3% | 18/96 (18.8) | Reference | Reference | Reference |
| **HER2‑positive** | 34 | 8.4 (6.8–10.1) | 4.3 (3.2–5.5) | 33.0% | 7/34 (20.6) | 0.98 (0.68–1.39); 0.91 | 1.02 (0.72–1.45); 0.92 | 1.12 (0.51–2.36); 0.78 |
| **PD‑L1 status** |  |  |  |  |  |  |  |  |
| **CPS <1** | 73 | 8.2 (6.7–9.7) | 4.1 (3.2–5.0) | 31.0% | 13/73 (17.8) | Reference | Reference | Reference |
| **CPS ≥1** | 57 | 8.5 (7.0–10.0) | 4.2 (3.4–5.2) | 32.5% | 12/57 (21.1) | 0.95 (0.71–1.28); 0.74 | 1.01 (0.75–1.36); 0.94 | 1.21 (0.62–2.35); 0.58 |
| **MSI status** |  |  |  |  |  |  |  |  |
| **Non‑MSI‑H** | 119 | 8.3 (7.0–9.7) | 4.2 (3.3–5.1) | 31.6% | 23/119 (19.3) | Reference | Reference | Reference |
| **MSI‑H** | 11 | 9.1 (6.0–13.0) | 4.5 (3.0–6.5) | 34.5% | 2/11 (18.2) | 0.90 (0.48–1.70); 0.74 | 0.94 (0.50–1.74); 0.86 | 0.93 (0.23–3.67); 0.92 |

† **Adjusted HRs** from Cox models controlling for **age, sex, ECOG (0–1 vs ≥2)**, and **NLR (<3 vs ≥3)**; **HER2 models** additionally adjust for **prior trastuzumab; PD‑L1/MSI‑H models** adjust for **prior ICI**.

‡ **Adjusted ORs** for **ORR** from logistic regression with the same covariates.

**Supplementary Table S2. Outcomes by prior therapy exposure**

| **Group** | **n** | **Median OS, mo (95% CI)** | **Median PFS, mo (95% CI)** | **6‑mo OS (%)** | **12‑mo OS (%)** | **ORR n/N (%)** | **DCR n/N (%)** | **Adjusted HR for OS† (95% CI); p** | **Adjusted HR for PFS† (95% CI); p** | **Adjusted OR for ORR‡ (95% CI); p** |
| --- | --- | --- | --- | --- | --- | --- | --- | --- | --- | --- |
| **No prior ICI** | 116 | 8.4 (7.0–9.9) | 4.2 (3.3–5.2) | 54.7 | 31.9 | 22/116 (19.0) | 58/116 (50.0) | Reference | Reference | Reference |
| **Prior ICI (any PD‑1/PD‑L1/CTLA‑4)** | 14 | 8.0 (5.4–10.2) | 3.9 (2.6–5.1) | 53.6 | 30.0 | 3/14 (21.4) | 7/14 (50.0) | 0.96 (0.58–1.59); 0.86 | 1.04 (0.65–1.66); 0.86 | 1.15 (0.33–3.09); 0.84 |

**Supplementary Table S3. Outcomes by prior therapy exposure**

|  | **n** | **Median OS, mo (95% CI)** | **Median PFS, mo (95% CI)** | **6‑mo OS (%)** | **12‑mo OS (%)** | **ORR n/N (%)** | **DCR n/N (%)** | **Adjusted HR for OS† (95% CI); p** | **Adjusted HR for PFS† (95% CI); p** | **Adjusted OR for ORR‡ (95% CI); p** |
| --- | --- | --- | --- | --- | --- | --- | --- | --- | --- | --- |
| **No prior trastuzumab** | 16 | 8.1 (6.0–10.1) | 4.0 (2.9–5.4) | 52.5 | 29.4 | 3/16 (18.8) | 8/16 (50.0) | Reference | Reference | Reference |
| **Prior trastuzumab** | 18 | 8.5 (6.5–10.6) | 4.4 (3.0–5.9) | 55.6 | 33.3 | 4/18 (22.2) | 9/18 (50.0) | 0.92 (0.56–1.52); 0.75 | 0.95 (0.59–1.52); 0.82 | 1.24 (0.30–5.05); 0.78 |

† **Adjusted HRs** from Cox models for OS/PFS controlling for **age, sex, ECOG (0–1 vs ≥2)**, and **NLR (<3.0 vs ≥3.0)**;

‡ **Adjusted ORs** from logistic regression for **ORR** using the same covariates.
